# The Presence of Added Sugars and Other Sweeteners in Food and Beverage Products Advertised on Television in the United States, 2022

**DOI:** 10.3390/nu16233981

**Published:** 2024-11-21

**Authors:** Rebecca M. Schermbeck, Julien Leider, Lisa M. Powell

**Affiliations:** 1 Institute for Health Research and Policy, University of Illinois Chicago, Chicago, IL 60608, USA; 2 Division of Health Policy and Administration, School of Public Health, University of Illinois Chicago, Chicago, IL 60612, USA

**Keywords:** added sugars, artificial sweeteners, sugar, sugar alcohols, natural sweeteners, advertisements, children, adolescents, adults

## Abstract

Background/Objectives: The Dietary Guidelines for Americans recommend consuming less than 10% of total calories from added sugars. Low-calorie sweeteners, sugar alcohols, and natural low-calorie sweeteners are used to reduce added sugar intake, but there are concerns about their long-term health impacts, especially for children. This paper describes the food and beverage television advertising landscape as it pertains to sweeteners. Methods: This cross-sectional study uses television ratings data licensed from The Nielsen Company for the United States in 2022. Nutrition facts panels and ingredient lists were collected for food and beverage product advertisements seen on television and assessed for the presence of added sugars, low-calorie sweeteners, sugar alcohols, and natural low-calorie sweeteners (forms of stevia and monk fruit), as well as whether products were high in added sugars based on federal Interagency Working Group guidelines for advertising to children. Results: Of the sweeteners examined, added sugars were most commonly found in food and beverage product advertisements seen on television (60–68% of advertisements seen across age groups), followed by low-calorie sweeteners (6–10%), sugar alcohols (2–4%), and natural low-calorie sweeteners (2%). About one-third (32–33%) of advertisements seen by 2–5- and 6–11-year-olds were high in added sugars, similar to the percentage seen by 12–17- and 18+-year-olds (34–35%). Advertisements seen for cereal (86–95%) and sweets (92–93%) were most likely to have added sugars, while those for sweets (89–90%) were most likely to be high in added sugars. Conclusions: Sweeteners are common in food and beverage product advertisements seen on television, including alternatives to added sugars for which there are concerns about long-term impacts on health. Continued monitoring and additional research on other advertising media platforms used by food and beverage companies (e.g., digital media) is needed.

## 1. Introduction

The 2020–2025 Dietary Guidelines for Americans recommend that less than 10% of total calories come from added sugars. Yet, added sugars account for more than 13% of the average American’s calorie intake [1]. While adding sweeteners like sugar or honey to a food or beverage is an obvious added sugar, most people consume added sugars from processed and packaged food and beverage products like sugar-sweetened beverages, sweets and desserts, sweetened coffee and tea, and candy [1]. These products are calorically dense with minimal nutritional value and contribute to a host of health problems, including obesity, type 2 diabetes, and heart disease [2]. Consumers may not be entirely aware of what is in their processed and packaged foods and beverages, as reading ingredient lists can be difficult [3] and manufacturers utilize a variety of sweeteners aside from sugar that contribute added sugars [4].

In 2016, the Food and Drug Administration (FDA) updated the nutrition facts panel to help consumers make more informed decisions about the foods and beverages they consume. The updated panel added a new line of information about added sugars present in a product. Large food and beverage companies (annual sales of USD 10 million or more) had until 1 January 2020, and smaller companies (annual sales less than USD 10 million) had until 1 January 2021, to update nutrition labels [5]. The inclusion of added sugars on the nutrition panel is expected to have a positive impact on health care costs and diseases associated with overconsumption of added sugars [6].

Reducing consumption of added sugars may also be achieved by replacing added sugars with low-calorie, nonnutritive sweeteners. Nonnutritive sweeteners can be anywhere from 200 to 20,000 times sweeter than table sugar [7]. The FDA has approved six low-calorie, high-intensity sweeteners (saccharine, aspartame, acesulfame potassium, sucralose, neotame, and advantame) as food additives. Steviol glycosides from the stevia plant and extracts from monk fruit are low-calorie sweeteners allowable for use due to the substances being “generally recognized as safe” (GRAS). Products are classified as GRAS based on scientific and public knowledge and can be used by companies without FDA approval. Sugar alcohols are another class of nonnutritive sweeteners available for use in food and beverages. Sugar alcohols include, for example, sorbitol, xylitol, lactitol, mannitol, erythritol, and maltitol [8]. Sales of food and beverage products with nonnutritive sweeteners have increased over the past two decades in tandem with a decrease in sales of products with caloric sweeteners [9].

Current literature is inconclusive as to the health impacts associated with nonnutritive sweetener consumption. A randomized controlled trial among adults with overweight or obesity found differences across nonnutritive sweeteners; saccharine consumption led to significant weight gain, whereas sucralose consumption led to decreased energy intake [10]. A systematic review with meta-analyses suggested the use of nonnutritive sweeteners in place of sugar may lead to reduced energy intake and body weight [11]. In one large cohort study, nonnutritive sweeteners (especially aspartame and acesulfame-K) were associated with increased risk of cancer [12]. A systematic review and meta-analysis found that nonnutritive sweetener consumption does not impact blood glucose levels [13], although results of at least one study suggested consumption may increase the risk of glucose intolerance in animals and humans and increase the risk of type 2 diabetes in humans [14]. Two studies have reported evidence that the consumption of the sugar alcohol erythritol negatively impacts blood clotting function and cardiovascular health [15,16]. More research is needed on the long-term health impacts of consuming low-calorie, nonnutritive sweeteners [10,11,12,13,14,15,16,17].

Recommendations regarding nonnutritive sweetener consumption vary, including by age. The Academy of Nutrition and Dietetics, the American Heart Association, and the American Diabetes Association have stated that nonnutritive sweeteners, when consumed judiciously, could be helpful in reducing caloric consumption, weight gain, and associated medical conditions for adults [18,19]. However, the Dietary Guidelines for Americans note that nonnutritive sweeteners “may not be a good way to manage your weight in the long run” [1]. The World Health Organization also recommends nonnutritive sweeteners not be used as a way to control weight and related diseases [20]. For children specifically, the American Heart Association Scientific Advisory Board released a statement advising against children consuming nonnutritive sweeteners [21]. Additionally, the American Academy of Pediatrics has stated that long-term safety has not been adequately assessed for children and suggests nonnutritive sweetener information should be added to the nutrition facts panel so parents can make more informed decisions on purchases [17].

Food and beverage manufacturers spent USD 4.0 billion on television advertisements in 2021 [22]. Data from 2022 show over 60% of advertisements seen by 2–5- and 6–11-year-old children for food and beverage products on all programming and children’s programming were for products high in saturated fat, trans fat, sodium, and/or sugars [23]. This recent research did not assess if food and beverage products advertised on television contained nonnutritive sweeteners. Identifying and monitoring food and beverage products advertised on television using nonnutritive sweeteners is warranted given increased sales [9], unknown long-term health effects [10,11,12,13,14,15,16,17], and variation in recommendations from the government and leading health authorities [1,18,19,20].

There are no formal government regulations in the United States (U.S.) on what foods and beverages are allowable to advertise on all programming or children’s programming with regards to nutrition in general and specifically added sugars and nonnutritive sweeteners. The Children’s Food and Beverage Advertising Initiative (CFBAI) is a voluntary organization started in 2006 with company-specific guidelines for food and beverage advertising directed to children on television and other media [24] that evolved in 2014, with further changes in 2020, to include uniform nutrition criteria that all companies choosing to be part of the initiative must follow. The guidelines include 17 different food and beverage product categories with limits on calories, saturated fat, sodium, and added sugars [25]. Currently, there are 21 food and beverage manufacturer members [26].

In 2009, representatives from the Federal Trade Commission, the Centers for Disease Control and Prevention, the FDA, and the U.S. Department of Agriculture (USDA) formed an Interagency Working Group (IWG) to develop guidelines for foods and beverages marketed to children on television and other forms of media, including limits of no more than 13 g of added sugars per reference amount customarily consumed for individual foods and per serving for main dishes and meals [27]. Neither the CFBAI nor the IWG have addressed nonnutritive sweeteners, and both guidelines are voluntary. The objective of this paper is to describe the food and beverage television advertising landscape as it pertains to sweeteners in foods and beverages advertised on television, specifically added sugars, low-calorie sweeteners, sugar alcohols, and natural low-calorie sweeteners.

## 2. Materials and Methods

Cross-sectional television ratings data for 2022 on the percentage of individuals by age group (2–5, 6–11, 12–17, and 18+ years old) in U.S. television households that saw a program or advertisement were licensed from The Nielsen Company. The television ratings data used in this study covered exposure to non-Spanish language broadcast and cable network, syndicated, and spot television advertising, including from both all programming and specifically children’s programming, defined as programs where the child (2–11 years) audience share is 35% or greater. Data were also obtained by self-reported race, which was collected during periodic personal interviews conducted by Nielsen; the data by race did not include exposure to spot television advertising. The data provided by The Nielsen Company included the number of advertisements seen by product, program, and channel, aggregated across their national sample of television-equipped households (41,000 households on average); the data were weighted to ensure representativeness. Further methodological details can be found elsewhere [23].

Food and beverage products were classified as beverages, cereal, snacks (e.g., pretzels, chips, crackers), sweets (e.g., candy, frozen novelties, cookies), and other foods (e.g., canned beans, frozen pizza, soup). Nutrition facts panels and ingredient lists were obtained from the USDA’s Food Data Central, manufacturer websites, and product food labels (obtained from grocery store websites or in-person). We were unable to obtain relevant nutrition information for 11% or less of food and beverage product advertisements seen across sweeteners, age groups, and programming audiences. Information could not be obtained if the product was a nonspecific food product (e.g., Dairy Association or general food company advertisement) or the sources above did not have the information. Nutrition information for added sugars, when available, was collected for each food and beverage product. Each food and beverage product’s ingredient list was reviewed for the presence of low-calorie sweeteners (acesulfame potassium, aspartame, saccharine, sucralose, neotame, and advantame), sugar alcohols (sorbitol, xylitol, lactitol, mannitol, erythritol, and maltitol), and natural low-calorie sweeteners (forms of stevia and monk fruit) [8].

Each food and beverage product’s nutrition facts panel was reviewed for added sugars. If present, the product was identified as having added sugars. A food or beverage product was categorized as high in added sugars if it exceeded the IWG’s recommended limit of 13 g of added sugars per reference amount customarily consumed for individual foods (e.g., candy, cookies, yogurt, and cereal) or 13 g of added sugars per serving for main dishes (e.g., frozen pizza, canned soup) and meals (e.g., frozen dinners) [27]. For food and beverage products that did not have added sugars on the nutrition label, researchers coded added sugars as 0 g when there were 0 g total sugars listed on the label or the ingredient list contained no caloric sweeteners. This process allowed researchers to identify whether products were high in added sugars for at least 95% of the food and beverage product advertisements seen across age groups and programming audiences. There are not labeling requirements or recommended limits from the IWG or CFBAI for low-calorie sweeteners, sugar alcohols, or natural low-calorie sweeteners. Gram amounts of these sweeteners are generally not available on nutrition fact panels. Thus, the ingredient list of each product was reviewed for the presence of the low-calorie sweeteners, sugar alcohols, or natural low-calorie sweeteners listed above. Descriptive statistics were computed in Stata/MP 18.0 [28].

## 3. Results

Table 1 shows the percentage of advertisements seen on all programming with each sweetener type, by age group and product category. Added sugars were the most commonly found sweetener in food and beverage product advertisements seen on television (60–68% of advertisements seen across age groups), followed by low-calorie sweeteners (6–10%), sugar alcohols (2–4%), and natural low-calorie sweeteners (2%). Overall, 8–14% of advertisements seen had either low-calorie sweeteners, sugar alcohols, or natural low-calorie sweeteners. More food and beverage advertisements seen by 2–5-, 6–11-, and 12–17-year-olds had added sugars (66–68%) compared to advertisements seen by 18+-year-olds (60%). Substantial percentages of advertisements seen for all product categories had added sugars, but prevalence was highest for cereal (86–95% of advertisements seen across age groups) and sweets (92–93%).

Roughly one-third of the food and beverage product advertisements seen by children (32.0% for 2–5-year-olds, 32.9% for 6–11-year-olds) exceeded the IWG recommendations for products with added sugars; by way of comparison, this figure was 34.7% for 12–17-year-olds and 33.8% for 18+-year-olds. Almost 9 in 10 (89–90%) of advertisements seen for sweets were high in added sugars.

Advertisements for food and beverages with low-calorie sweeteners, sugar alcohols, and natural low-calorie sweeteners were seen on television much less than advertisements for products with added sugars. Specifically, 9.6%, 9.7%, 8.7%, and 5.6% of the food and beverage product advertisements seen on television by ages 2–5 years, 6–11 years, 12–17 years, and 18+ years, respectively, were for products with low-calorie sweeteners. The use of sugar alcohols as a sweetener in food and beverage product advertisements seen on television was low generally but particularly for older age groups (4.4% for 2–5-year-olds, 4.2% for 6–11-year-olds, 3.5% for 12–17-year-olds, and 1.9% for 18+-year-olds). Natural low-calorie sweeteners were identified in food and beverage product advertisements seen even less than sugar alcohols (1.8% for 2–5-year-olds, 1.7% for 6–11-year-olds, 2.0% for 12–17-year-olds, 2.3% for 18+-year-olds).

There were differences in food and beverage product advertisements seen on television based on companies’ participation in CFBAI (Table 2). About 75% of food and beverage product advertisements seen by children 2–11-years old on all programming from CFBAI companies contained added sugars, compared to 45% of food and beverage product advertisements seen from non-CFBAI companies. On children’s programming, the percentage of food and beverage product advertisements seen containing added sugars was 100.0% for CFBAI company advertisements seen by 2–11-year-olds, 48.4% for 2–5-year-olds, and 55.7% for 6–11-year-olds for non-CFBAI companies. Children saw a greater percentage of food and beverage advertisements high in added sugars from CFBAI companies compared to non-CFBAI companies on all programming (36.3% vs. 22.1% for 2–5-year-olds; 36.4% vs. 23.8% for 6–11-year-olds). However, the percentage of food and beverage product advertisements seen that were high in added sugars was only roughly 12% on children’s programming for both age groups from CFBAI companies and for 2–5-year-olds from non-CFBAI companies. Children saw a higher percentage of food and beverage product advertisements with low-calorie sweeteners from CFBAI companies (12.9–28.5%) compared to non-CFBAI companies (1.9–3.6%) across both age groups and both all and children’s programming. Specifically on children’s programming, children 2–11-years old saw a higher percentage of food and beverage product advertisements containing low-calorie sweeteners (28.4–28.5%) from CFBAI companies compared to advertisements for food and beverage products high in added sugars (12.1–12.4%). The food and beverage product advertisements seen from non-CFBAI companies contained sugar alcohols and natural low-calorie sweeteners more often than those seen from CFBAI companies. For example, 11.2–24.5% of food and beverage product advertisements seen by children from non-CFBAI companies contained at least one sugar alcohol, compared to 0.0–1.3% of CFBAI food and beverage advertisements seen by children.

Table 3 shows the percentage of food and beverage product advertisements seen on all programming with each sweetener type, by race and age group. The percentage of advertisements seen with each sweetener type was generally similar by race.

## 4. Discussion

Governments across the globe have implemented a number of different types of policies (e.g., sugar-sweetened beverage taxes, healthy checkout ordinances, and enhanced labeling requirements) with the aim of improving population health, including a reduction in added sugar purchases and consumption [29,30,31]. Industry has responded in part by reformulating products to use nonnutritive sweeteners [32], but the long-term health implications of increased consumption of nonnutritive sweeteners are not clear [10,11,12,13,14,15,16,17]. Knowing the distribution of the types of sweeteners used in food and beverage products that are being promoted is important and should be monitored.

This cross-sectional study examined the sweeteners (added sugars, low-calorie sweeteners, sugar alcohols, and natural low-calorie sweeteners) used in food and beverage product advertisements seen on television. Of all the sweeteners considered, added sugars were most often present in food and beverage product advertisements seen on all television programming, regardless of age. Advertisements for food and beverage products containing low-calorie sweeteners, sugar alcohols, and natural low-calorie sweeteners were also seen, but much less often than for products with added sugars. In particular, this study found only 2% of advertisements seen were for products containing natural low-calorie sweeteners (forms of stevia and monk fruit).

While from 2002 to 2018, food and beverage products containing only caloric sweeteners continued to be purchased by essentially all U.S. households (99.9–100%), there was a statistically significant increase in the percentage of households purchasing food and beverage products containing both caloric sweeteners and nonnutritive sweeteners (from 46.7% to 74.1%) as well as in the percentage purchasing products containing only nonnutritive sweeteners (from 65.7% to 67.2%). The percentage of households purchasing beverages containing a combination of caloric and nonnutritive sweeteners increased from 15.9% to 49.4% [9].

The use of nonnutritive sweeteners in food and beverage products varies greatly worldwide. In Chile, researchers conducted a study to identify the percentage of food and beverage products available in supermarkets containing nonnutritive sweeteners. Researchers collected nutrition information from December 2018 through October 2019 for 1489 food and beverage products and found that 815 (55.5%) of the products contained at least one nonnutritive sweetener, with sucralose being the most prevalent, followed by steviol glycosides [33]. Chile is reporting some of the highest proportions of food and beverages containing nonnutritive sweeteners compared to other countries. Dunford et al. (2018) used country-specific nutrition databases to collect nonnutritive sweetener information on food and beverage products in Mexico, Australia, New Zealand, and the U.S. and found that Mexico had the highest proportion (11.08%) of products containing at least one nonnutritive sweetener, followed by the U.S. (4.37%), New Zealand (1.44%), and Australia (0.86%) [34].

In another study examining the presence of nonnutritive sweeteners in food and beverage products, researchers reported that in 2015, free or added sugars were present in 62.7% of food and beverage products available in Australian supermarkets. The percentage of free or added sugars present decreased to 59.9% of food and beverages available in Australian supermarkets in 2019, but these remained the most commonly used sweetener. Non-nutritive sweeteners (aspartame, saccharine, sucralose, cyclamate, acesulfame K, steviol glycosides, neotame, thaumatin, alitame, and monk fruit extract) and sugar alcohols used as sweeteners were found in 3.8% and 2.3% of food and beverage products available in Australian supermarkets in 2015. These percentages increased slightly to 4.3% and 3.4% in 2019 for nonnutritive sweeteners and sugar alcohols, respectively [35].

The landscape of food and beverage advertising to children on television has changed since the CFBAI began. A number of companies that are part of the initiative have pledged to no longer advertise foods and beverages on children’s television programming [26]. Indeed, a recent study showed that children’s exposure to food and beverage product advertisements on children’s programming fell substantially from 2013 to 2022; however, the majority of CFBAI-member product advertisements seen by children still do not meet the IWG nutrition recommendations [23].

In this study, 12% of advertisements seen on children’s programming from CFBAI companies were for products high in added sugars as defined by the IWG. The CFBAI limits added sugars but not low-calorie sweeteners, and indeed this study found that children saw a smaller percentage of food and beverage advertisements for products high in added sugars from CFBAI companies compared to products containing low-calorie sweeteners from CFBAI companies on children’s programming. This was not true of CFBAI companies for all programming, but the industry [25] and IWG nutrition guidelines [27] only pertain to children’s programming. Therefore, it is important to consider limiting children’s exposure to unhealthy product advertising from all sources of programming on television. Additionally, future research should examine other forms of media, as exposure to television advertising of food and beverage products has decreased significantly since 2017 [22,23].

This study provides important insights through the use of a nationally representative sample of data covering advertising exposure from both cable and network television. Nonetheless, it is subject to some limitations. First, because analyses relied on aggregated data from Nielsen, it was not possible to account for sampling variability or assess the statistical significance of differences in advertising exposure. Second, because of a lack of detailed information on low-calorie sweeteners, sugar alcohol, and natural low-calorie sweetener content on nutrition facts panels, this study is limited to assessing whether products seen included these sweeteners and could not assess the amount of these sweeteners.

## 5. Conclusions

Added sugars are the most common sweetener in food and beverage product advertisements seen on television, followed by low-calorie sweeteners. A small percentage of food and beverage product advertisements seen on television use sugar alcohols, and natural low-calorie sweeteners as sweeteners. Recent trends indicate an increase in the presence of nonnutritive sweeteners in food and beverage products available worldwide. The research on low-calorie sweeteners, sugar alcohols and natural low-calorie sweeteners used in food and beverage products and the impact of consumption on long-term health is growing, but the long-term health effects of consumption of these products are still unknown and of concern, especially for children. More transparency on nutrition facts panels would be helpful to both inform consumers and provide information needed to better assess the nutritional content of food and beverage products marketed to children and adults. Researchers should continue to monitor the sweeteners found present in product advertisements on television and also examine their presence in other media sources.

## Figures and Tables

**Table 1:** Percentage of food and beverage product television advertisements seen on all programming with added sugars, high in added sugars, and with low-calorie sweeteners, sugar alcohols, and natural low-calorie sweeteners by age group and product category, 2022.

	Have Added Sugars (%)	High in Added Sugars (%)	Have Other Sweeteners ^1^ (%)	Have Low-Calorie Sweeteners ^2^ (%)	Have Sugar Alcohols ^3^ (%)	Have Natural Low-Calorie Sweeteners ^4^ (%)
Age Group & Product Category						
2–5 years						
All Food and Beverage Products	67.0	32.0	14.1	9.6	4.4	1.8
Beverages	49.6	32.5	17.2	14.2	0.1	3.8
Cereal	94.0	20.7	3.2	0.0	0.0	3.2
Snacks	40.5	7.4	3.2	2.2	1.8	1.0
Sweets	92.4	89.6	17.3	5.8	17.3	0.0
Other	50.0	5.7	19.7	18.2	0.1	1.5
6–11 years						
All Food and Beverage Products	68.0	32.9	14.0	9.7	4.2	1.7
Beverages	49.9	33.7	17.7	14.7	0.1	3.8
Cereal	94.8	21.1	2.7	0.0	0.0	2.7
Snacks	40.0	7.4	2.0	1.2	0.9	0.8
Sweets	92.5	89.8	16.7	5.6	16.7	0.0
Other	50.5	6.3	21.4	19.8	0.1	1.6
12–17 years						
All Food and Beverage Products	65.7	34.7	12.3	8.7	3.5	2.0
Beverages	51.3	36.6	18.2	14.9	0.1	4.1
Cereal	93.0	20.6	3.7	0.0	0.0	3.7
Snacks	39.8	7.0	2.0	1.1	0.7	1.0
Sweets	91.5	88.9	13.4	6.6	13.4	0.0
Other	47.2	6.9	16.0	14.2	0.1	1.7
18+ years						
All Food and Beverage Products	59.5	33.8	8.0	5.6	1.9	2.3
Beverages	47.6	29.7	16.4	13.4	0.1	3.9
Cereal	86.2	20.3	7.2	0.0	0.0	7.2
Snacks	39.8	7.6	1.8	1.1	0.8	0.7
Sweets	92.8	88.9	6.9	5.9	6.9	0.0
Other	36.7	7.9	6.1	3.9	0.2	2.2

Source: Data were licensed from The Nielsen Company. ^1^ Other sweeteners included low-calorie sweeteners, sugar alcohols, and natural low-calorie sweeteners. ^2^ Low-calorie sweeteners included acesulfame potassium, aspartame, saccharine, sucralose, neotame, and advantame. ^3^ Sugar alcohols included sorbitol, xylitol, lactitol, mannitol, erythritol, and maltitol. ^4^ Natural low-calorie sweeteners included forms of stevia and monk fruit.

**Table 2:** Percentage of Food and Beverage Product Television Advertisements Seen on All and Children’s Programming with Added Sugars, High in Added Sugars, and with Low-calorie Sweeteners, Sugar Alcohols, and Natural Low-calorie Sweeteners by Age Group and CFBAI ^1^ Status, 2022.

	Have Added Sugars		High in Added Sugars		Have Low-Calorie Sweeteners ^2^		Have Sugar Alcohols ^3^		Have Natural Low-Calorie Sweeteners ^4^	
	Programming (%)		Programming (%)		Programming (%)		Programming (%)		Programming (%)	
	All	Child ^5^	All	Child ^5^	All	Child ^5^	All	Child ^5^	All	Child ^5^
2–5 years										
Overall	67.0	71.6	32.0	12.4	9.6	14.7	4.4	7.5	1.8	0.2
CFBAI	76.1	100.0	36.3	12.4	12.9	28.5	1.3	0.0	1.4	0.0
Non-CFBAI	44.5	48.4	22.1	12.4	2.4	3.6	11.2	13.5	2.7	0.4
6–11 years										
Overall	68.0	86.0	32.9	17.1	9.7	19.4	4.2	8.3	1.7	0.1
CFBAI	76.4	100.0	36.4	12.1	12.9	28.4	1.3	0.0	1.4	0.0
Non-CFBAI	44.4	55.7	23.8	28.2	2.1	1.9	11.3	24.5	2.5	0.2

Source: Data were licensed from The Nielsen Company. ^1^ CFBAI: Children’s Food and Beverage Advertising Initiative. ^2^ Low-calorie sweeteners included acesulfame potassium, aspartame, saccharine, sucralose, neotame, and advantame. ^3^ Sugar alcohols included sorbitol, xylitol, lactitol, mannitol, erythritol, and maltitol. ^4^ Natural low-calorie sweeteners included forms of stevia and monk fruit. ^5^ ≥35% child audience.

**Table 3:** Percentage of food and beverage product television advertisements seen on all programming with added sugars, high in added sugars, and with low-calorie sweeteners, sugar alcohols, and natural low-calorie sweeteners by race and age group, 2022.

	Have Added Sugars		High in Added Sugars		Have Low-Calorie Sweeteners ^1^		Have Sugar Alcohols ^2^		Have Natural Low-Calorie Sweeteners ^3^	
	White (%)	Black (%)	White (%)	Black (%)	White (%)	Black (%)	White (%)	Black (%)	White (%)	Black (%)
Age Group										
2–5 years	68.5	66.7	32.9	33.4	10.1	8.4	4.8	3.9	1.6	1.8
6–11 years	68.3	69.2	34.3	32.9	9.6	9.6	4.4	4.4	1.6	1.6
12–17 years	66.1	68.4	35.9	34.9	8.8	8.8	3.6	4.0	2.0	1.7
18+ years	60.5	61.3	34.7	35.3	5.9	5.7	1.9	1.9	2.2	2.2

Source: Data were licensed from The Nielsen Company. ^1^ Low-calorie sweeteners included acesulfame potassium, aspartame, saccharine, sucralose, neotame, and advantame. ^2^ Sugar alcohols included sorbitol, xylitol, lactitol, mannitol, erythritol, and maltitol. ^3^ Natural low-calorie sweeteners included forms of stevia and monk fruit.

## Data Availability

The Nielsen advertising data on which the analyses in this study rely cannot be shared per our contract with The Nielsen Company.
